# Supplementary material for: Enabling reactive microscopy with MicroMator
Source: Nat Commun. 2022 Apr 22;13:2199. doi: 10.1038/s41467-022-29888-z (PMC9033843; doi:10.1038/s41467-022-29888-z)
Supplement: Supplementary file 2 — Reporting Summary [file 41467_2022_29888_MOESM2_ESM.pdf]

nature research

Corresponding author(s): Gregory Batt
Last updated by author(s): Mar 21, 2022

Reporting Summary

Nature Research wishes to improve the reproducibility of the work that we publish. This form provides structure for consistency and transparency in reporting. For further information on Nature Research policies, see our Editorial Policies and the Editorial Policy Checklist.

Statistics

For all statistical analyses, confirm that the following items are present in the figure legend, table legend, main text, or Methods section.

n/a	Confirmed	
☐	☒	The exact sample size (n) for each experimental group/condition, given as a discrete number and unit of measurement
☒	☐	A statement on whether measurements were taken from distinct samples or whether the same sample was measured repeatedly
☒	☐	The statistical test(s) used AND whether they are one- or two-sided<br>*Only common tests should be described solely by name; describe more complex techniques in the Methods section.*
☒	☐	A description of all covariates tested
☒	☐	A description of any assumptions or corrections, such as tests of normality and adjustment for multiple comparisons
☐	☒	A full description of the statistical parameters including central tendency (e.g. means) or other basic estimates (e.g. regression coefficient) AND variation (e.g. standard deviation) or associated estimates of uncertainty (e.g. confidence intervals)
☒	☐	For null hypothesis testing, the test statistic (e.g. F, t, r) with confidence intervals, effect sizes, degrees of freedom and P value noted<br>*Give P values as exact values whenever suitable.*
☒	☐	For Bayesian analysis, information on the choice of priors and Markov chain Monte Carlo settings
☒	☐	For hierarchical and complex designs, identification of the appropriate level for tests and full reporting of outcomes
☒	☐	Estimates of effect sizes (e.g. Cohen's d, Pearson's r), indicating how they were calculated

Our web collection on statistics for biologists contains articles on many of the points above.

Software and code

Policy information about availability of computer code

Data collection	The MicroMator software, together with event definitions for representative experiments (Fig 2c, Fig. 3e and Supplementary Fig. 5), can be found online: https://gitlab.inria.fr/InBio/Public/micromator.
Data analysis	Data analysis code for experiments (Fig. 3e, 4d, and Supplementary Fig. 5), as well as a tutorial example (Supplementary Note 2) and a tutorial movie (Supplementary Movie 1), can be found at the same place.

For manuscripts utilizing custom algorithms or software that are central to the research but not yet described in published literature, software must be made available to editors and reviewers. We strongly encourage code deposition in a community repository (e.g. GitHub). See the Nature Research guidelines for submitting code & software for further information.

Data

Policy information about availability of data

All manuscripts must include a data availability statement. This statement should provide the following information, where applicable:

- Accession codes, unique identifiers, or web links for publicly available datasets
- A list of figures that have associated raw data
- A description of any restrictions on data availability

Raw and processed data for Supplementary Fig. 1 (tutorial) and Fig. 2, and for Fig. 3c-e, 4c-d and Supplementary Fig. 5 are freely available on zenodo repositories: https://doi.org/10.5281/zenodo.5761545 (23GB) and https://doi.org/10.5281/zenodo.4616659 (45GB).

Field-specific reporting

Please select the one below that is the best fit for your research. If you are not sure, read the appropriate sections before making your selection.

☒ Life sciences ☐ Behavioural & social sciences ☐ Ecological, evolutionary & environmental sciences

For a reference copy of the document with all sections, see nature.com/documents/nr-reporting-summary-flat.pdf

Life sciences study design

All studies must disclose on these points even when the disclosure is negative.

Sample size	All cells in the field of view of the microscope have been considered in the study. Temporal resolution is 3 or 6 minutes as indicated in the text.
Data exclusions	A few cell trajectories have been discarded from the analysis, either based on physical localization (eg, cells at the border of field of view) or based on simple quality criteria (eg, very short trajectories). This is documented in a dedicated section of the supplementary material. These exclusions were motivated by known issues with such microfluidic microscopy experiments (eg cells sticking too close to the entrance of the flow chamber or flowing away mid experiment, leading to short measurement trajectories). Such criteria were pre-established during the development phase of the experimental platform and performed consistently across all microscopy experiments presented in the manuscript.
Replication	Video microscopy data include several tens (beginning of experiment) to several hundreds (end of experiment) of cells. We replicated each experiment presented in the manuscript at least twice with similar results. After establishing successful experimental conditions all experiments were able to be replicated, with the exception of a few external issues (power cuts to machinery while experiments were running, etc).
Randomization	We worked with yeast and bacterial cell cultures, in which cells were chosen at random from colonies to use in the study.
Blinding	Our results are proof of concepts that are not based on hypothesis testing studies. Blinding does not apply here.

Reporting for specific materials, systems and methods

We require information from authors about some types of materials, experimental systems and methods used in many studies. Here, indicate whether each material, system or method listed is relevant to your study. If you are not sure if a list item applies to your research, read the appropriate section before selecting a response.

Materials & experimental systems

n/a	Involved in the study
☒	☐ Antibodies
☒	☐ Eukaryotic cell lines
☒	☐ Palaeontology and archaeology
☒	☐ Animals and other organisms
☒	☐ Human research participants
☒	☐ Clinical data
☒	☐ Dual use research of concern

Methods

n/a	Involved in the study
☒	☐ ChIP-seq
☒	☐ Flow cytometry
☒	☐ MRI-based neuroimaging
