# Supplementary material for: Enabling reactive microscopy with MicroMator
Source: Nat Commun. 2022 Apr 22;13:2199. doi: 10.1038/s41467-022-29888-z (PMC9033843; doi:10.1038/s41467-022-29888-z)
Supplement: Supplementary file 4 — Description of Additional Supplementary Files [file 41467_2022_29888_MOESM4_ESM.pdf]

Title: Supplementary Movie 1:
Description: Launching_a_reactive_experiment_with_MicroMator.mp4. Screen capture movie showing all the steps needed to launch a MicroMator experiment.

Title: Supplementary Movie 2:
Description: real-time_segmentation_and_tracking_with_SegMator.mp4. Time-lapse movie showing the real-time segmentation and tracking quality obtained with SegMator. Left: bright-field image. Right: bright-field image overlaid with segmentation mask in cyan.

Title: Supplementary Movie 3:
Description: optogenetic_control_of_gene_expression-Open_loop.mp4. Time-lapse movie showing the response of the cells (mScarletI fluorescence) in an open-loop control experiment. Corresponds to Fig. 3c. Note that in this and subsequent movies, a rectangular region (the topmost 10%) is not illuminated for fluorescence since our DMD did not cover the entire region recorded by the camera.

Title: Supplementary Movie 4:
Description: ptogenetic_control_of_gene_expression-Population_closed_loop.mp4. Time-lapse movie showing the response of the cells (mScarletI fluorescence) in a population closed-loop control experiment. Corresponds to Fig. 3d.

Title: Supplementary Movie 5:
Description: optogenetic_control_of_gene_expression-Single_cell_closed_loop.mp4. Time-lapse movie showing the response of the cells (mScarletI fluorescence) in a single-cell closed-loop control experiment. Corresponds to Fig. 3e.

Title: Supplementary Movie 6:
Description: single_cell_recombination-Islets.mp4. Time-lapse movie showing the light signal sent to cells in order to create small islets of recombined cells. Corresponds to Fig. 4c.

Title: Supplementary Movie 7:
Description: single_cell_recombination-Ring.mp4. (Left) Time-lapse movie showing the light signal sent to cells in order to recombine all cells that have been at one moment in a ring-like pattern. (Right) Image showing the recombined state of the cells at the end of the experiment. Corresponds to Fig. 4d.
